# *In vivo* evaluation of the pharmacokinetic interactions between almonertinib and rivaroxaban, almonertinib and apixaban

**DOI:** 10.3389/fphar.2023.1263975

**Published:** 2023-10-04

**Authors:** Zhi Wang, Ying Li, Xueru He, Yuhao Fu, Yajing Li, Xin Zhou, Zhanjun Dong

**Affiliations:** ^1^ Graduate School of Hebei Medical University, Shijiazhuang, China; ^2^ Department of Pharmacy, Hebei General Hospital, Shijiazhuang, China

**Keywords:** UPLC-MS/MS, almonertinib, rivaroxaban, apixaban, pharmacokinetics, drug-drug interactions

## Abstract

**Background:** Almonertinib, a third-generation epidermal growth factor receptor tyrosine kinase inhibitor (EGFR-TKI), is commonly used as a first-line treatment for non-small cell lung cancer (NSCLC) patients with EGFR T790M mutations. Rivaroxaban and apixaban are a selective, direct factor Xa inhibitor used to treat venous thromboembolism (VTE), which is a frequent complication of NSCLC. Rivaroxaban and apixaban are substrates of CYP3A4, P-gp and BCRP, whereas almonertinib is an inhibitor of P-gp and BCRP. Rivaroxaban or apixaban are often prescribed together with almonertinib in NSCLC patients, but clear information on pharmacokinetic drug interaction is lacking. Therefore, this study aimed to unravel the extent of interactions between almonertinib-rivaroxaban and almonertinib apixaban in rats, and whether the pharmacokinetic interaction can be mitigated by rivaroxaban and apixaban dose adjustment.

**Methods:** Rats were divided into ten groups (*n* = 6) that received rivaroxaban (2 mg/kg) (group 1), apixaban (0.5 mg/kg) (group 2), almonertinib (15 mg/kg) (group 3, group 4), almonertinib with rivaroxaban (2 mg/kg) (group 5), almonertinib with rivaroxaban (1 mg/kg) (group 6), almonertinib with apixaban (0.5 mg/kg) (group 7), almonertinib with apixaban (0.25 mg/kg) (group 8), rivaroxaban (2 mg/kg) with almonertinib (group 9), apixaban (0.5 mg/kg) with almonertinib (group 10). The concentrations of drugs were determined by an ultra-performance liquid chromatography tandem mass spectrometry (UPLC-MS/MS). The levels of messenger RNA were determined using quantitative real-time polymerase chain reaction (qRT-PCR).

**Results and Discussion:** The results indicate that almonertinib increased the C_max_ and AUC_0-t_ of 2 mg/kg rivaroxaban by 3.30 and 3.60-fold, 1 mg/kg rivaroxaban by 1.28 and 1.90-fold. Almonertinib increased the C_max_ and AUC_0-t_ of 0.5 mg/kg apixaban by 2.69 and 2.87-fold, 0.25 mg/kg apixaban by 2.19 and 2.06-fold. In addition, rivaroxaban also increased systemic exposure to almonertinib. The results of qRT-PCR showed that almonertinib reduced the expression of Cyp3a1 in liver and intestine, and Abcb1a, Abcg2 in intestine and kidney. The pharmacokinetic results suggest that it is important to take special care of the interactions of these drugs in clinical applications.

## 1 Introduction

Venous thromboembolisms (VTEs) are a common and clinically significant complication in cancer patients, contributing to their mortality and morbidity (Garcia-Escobar et al., 2021). Compared to non-cancer-related VTEs, cancer-related VTEs cause a higher risk of recurrent VTEs and major bleeding (Vedovati et al., 2019). Lung cancer, the second most common cancer and the leading cause of cancer deaths, with non-small cell lung cancer (NSCLC) accounting for about 85% of all cases, is associated with an increased risk of VTEs and other thromboembolic complications, with an incidence of one in six patients (Numico et al., 2005; Tagalakis et al., 2007; Sung et al., 2021). As such, prevention and treatment of thromboembolism are crucial in patients with lung cancer. Direct oral anticoagulants (DOACs) have been shown to be effective, safe, and more convenient compared to low molecular weight heparins (LMWHs) and vitamin K-antagonists (VKAs) (O'Connell et al., 2021; Wojtukiewicz et al., 2020). Consequently, DOACs are widely used as a first-line treatment for the prevention and treatment of VTEs in cancer patients without renal and/or hepatic impairment, and without genitourinary or gastrointestinal tumors, according to clinical guidelines (Martin et al., 2021). However, the use of DOACs is not without concerns when combined with anticancer drugs due to the potential drug-drug interactions (DDIs) (Alsubaie et al., 2021; Streiff et al., 2021).

Rivaroxaban and apixaban are direct anticoagulants taken orally that competitively inhibits both free and clot-bound Factor Xa, as well as prothrombinase activity, thereby preventing the blood clotting cascade (Mueck et al., 2014; Byon et al., 2019; Ajmal et al., 2021). After oral administration, the two drugs rapidly absorbed and reaches maximum plasma concentrations in approximately 2–4 h (Kubitza et al., 2005a; Kubitza et al., 2005b; Raghavan et al., 2009). Rivaroxaban has a dual mode of elimination, with two-thirds metabolized by the liver and one-third excreted by renal. It is metabolized by several cytochrome P450 (CYP) enzymes, including CYP3A4 and CYP2J2, as well as via CYP-independent mechanisms (Mueck et al., 2013). The main site of apixaban biotransformation is the O-demethylation or hydroxylation of the 3-piperidinone group, with metabolism mainly occurring via CYP3A4, but also by CYP1A2, CYC2J2, CYC2C8, CYC2C9 and CYC2C19 (Wang et al., 2010; Granger et al., 2011). Renal excretion accounts for approximately 27% of the total clearance of apixaban (Byon et al., 2019). Both *in vitro* and *in vivo* studies indicated that the two drugs are substrates of P-glycoprotein (P-gp) and breast cancer resistance protein (BCRP) (Weinz et al., 2009; Gnoth et al., 2011; Chang et al., 2017). Induction or inhibition of transporter and drug-metabolizing enzyme activity by concomitant drug administration or pathophysiologies can lead to changes in the pharmacokinetics of rivaroxaban and apixaban (Antoniou, 2015; Di Minno et al., 2017). These changes may result in increased or decreased plasma drug concentration and affect its efficacy and safety. Given this, it is necessary to assess the pharmacokinetics of rivaroxaban and apixaban in the presence and absence of drugs possible co-administered.

Almonertinib is a novel, irreversible, and selective third-generation epidermal growth factor receptor tyrosine kinase inhibitor (EGFR-TKI) (Nagasaka et al., 2021) that exerts inhibitory effects on tumor metastasis by covalently binding to the ATP site on the tyrosine kinase domain (Hojjat-Farsangi, 2014; Yang et al., 2020). AENEAS trial reported superior progression-free survival among locally advanced or metastatic NSCLC patients receiving almonertinib compared with those receiving gefitinib (Lu et al., 2022a). Additionally, previous research (Zhang et al., 2021) showed that almonertinib easily penetrates the blood-brain barrier and inhibits advanced NSCLC brain and spinal cord metastases. It was approved for the first-line treatment of advanced NSCLC patients with EGFR-TKI-sensitive genetic mutations by the National Medical Products Administration (NMPA) of China in 2021 based on the AENEAS trial (Lu et al., 2022b). Due to the high incidence of VTE induced by lung cancer, almonertinib, and DOACs are often prescribed together in clinical management. However, some studies have indicated that almonertinib is a substrate of CYP3A4 and P-gp, as well as an inhibitor of P-gp and BCRP (Wu et al., 2021; Liu et al., 2022a). Although almonertinib was introduced late in the market and the reality of the combination therapy is not clear, it has a high risk of drug interactions based on existing theories. In clinic, clinicians are confused about whether almonertinib and DOACs can be co-administered and whether dose adjustment is required. Therefore, it is imperative to investigate the potential pharmacokinetic interplay between almonertinib and DOACs, as well as the magnitude of such interplay, to facilitate their optimal co-administration in clinical practice. In this study we examined the extent of pharmacokinetic interaction between the P-gp inhibitor almonertinib and rivaroxaban or apixaban in rats, and whether the pharmacokinetic interaction can be mitigated by rivaroxaban and apixaban dose adjustment. Furthermore, we assessed the changes in messenger RNA of CYP3A4, P-gp, and BCRP in the liver, intestines and kidney of rats to clarify the potential mechanisms.

Several methods exist for determining the blood concentration of rivaroxaban and apixaban, but they each have limitations, such as a narrow range of calibration curves (Lagoutte-Renosi et al., 2018; Dunois, 2021), a high volume of plasma required, tedious pre-processing steps, and the use of a large among of organic reagents (de Oliveira et al., 2021; Zidekova et al., 2023). While a validated ultra-performance liquid chromatography-tandem mass spectrometry (UPLC-MS/MS) method is available for determining rivaroxaban and apixaban, it can not be used to quantify almonertinib under these conditions (Zhang et al., 2016). Similarly, several methods can be used to determine the blood concentration of almonertinib (Liu et al., 2022b; Li et al., 2022; Tang et al., 2022), but none are suitable for the simultaneous determination of rivaroxaban, apixaban and almonertinib in the plasma due to their characteristics and limitations. Therefore, in this study, a sensitive, simple, and rapid UPLC-MS/MS method was also developed and validated for pharmacokinetic interaction study.

## 2 Materials and methods

### 2.1 Chemicals and regents

**FIGURE 1:**
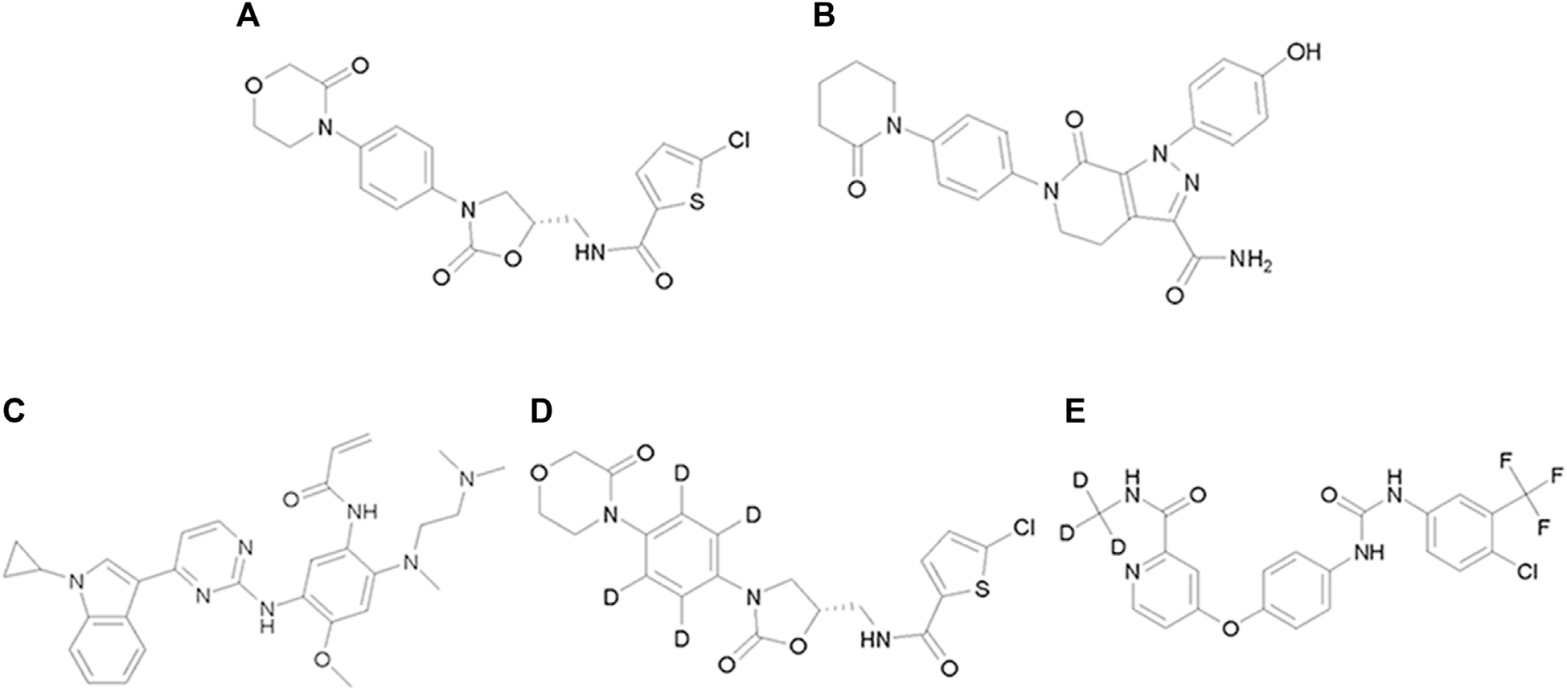
Chemical structure of rivaroxaban **(A)**, apixaban **(B)**, almonertinib **(C)**, rivaroxaban-d_4_ **(D)** and sorafenib-d_3_ **(E)**.

Almonertinib (purity>98%, Figure 1) was supplied by Jiangsu Hansoh Pharmaceutical Group Co., Ltd. (Lianyungang, China). Sorafenib-d_3_ (purity 99.5%, Lot ZZS-20-X261-A1, Figure 1) was purchased from Shanghai Zhen Zhun Biotechnology Co., Ltd. (Shanghai, China). Rivaroxaban (purity ≥ 99%, Lot H25J9Z64216, Figure 1) was provided by Shanghai yuan ye Bio-Technology Co. Ltd. (Shanghai, China). Apixaban (purity ≥ 98%, Lot C15069980, Figure 1) was purchased from Shanghai macklin Bio-Technology Co. Ltd. (Shanghai, China). Rivaroxaban-d_4_ (purity > 98%, Lot 21,702, Figure 1) was obtained from B1203 Life Science Park, SCT Creative Factory. (Shenzhen, China). Dimethyl sulfoxide (DMSO) was acquired from Beijing Solarbio Science Technology Co. Ltd. (Beijing, China). High-performance liquid chromatography (HPLC)-grade acetonitrile and formic acid were supplied by Fisher Scientific (Pittsburgh, PA, United States). Ultrapure water was used throughout the study and purchased from Wahaha Group Co., Ltd. (Hangzhou, China). The TRNzol Universal Reagent, FastKing RT Kit (with DNase), and SuperReal PreMix Plus (SYBR Green) were purchased from Tiangen Biotech Co., Ltd. (Beijing, China).

### 2.2 Instrumentation and chromatographic conditions

Analytes were measured using UPLC-MS/MS, which was equipped with an LC-30A ultra-performance liquid chromatography (Shimadzu, Kyoto, Japan) and Sciex Triple Quad 5,500 tandem triple quadrupole mass spectrometer (AB Sciex, Framingham, MA, United States). Chromatographic separation was performed using an XSelect HSS T3 column (2.1 mm × 100 mm, 2.5 µm, Waters, Milford, MA, United States) at 40°C by gradient elution. The mobile phase consisted of water (A) and acetonitrile (0.1% formic acid, B). The elution procedure was as follows: 0–1.0 min, 50% B; 1.0–1.5 min, 50%→98% B; 1.5–3.0 min, 98% B; 3.0–3.1 min, 98%→50% B; 3.1–4.1 min, 50% B. The flow rate was 0.35 mL min^−1^, and the injection volume was 6 µL.

**FIGURE 2:**
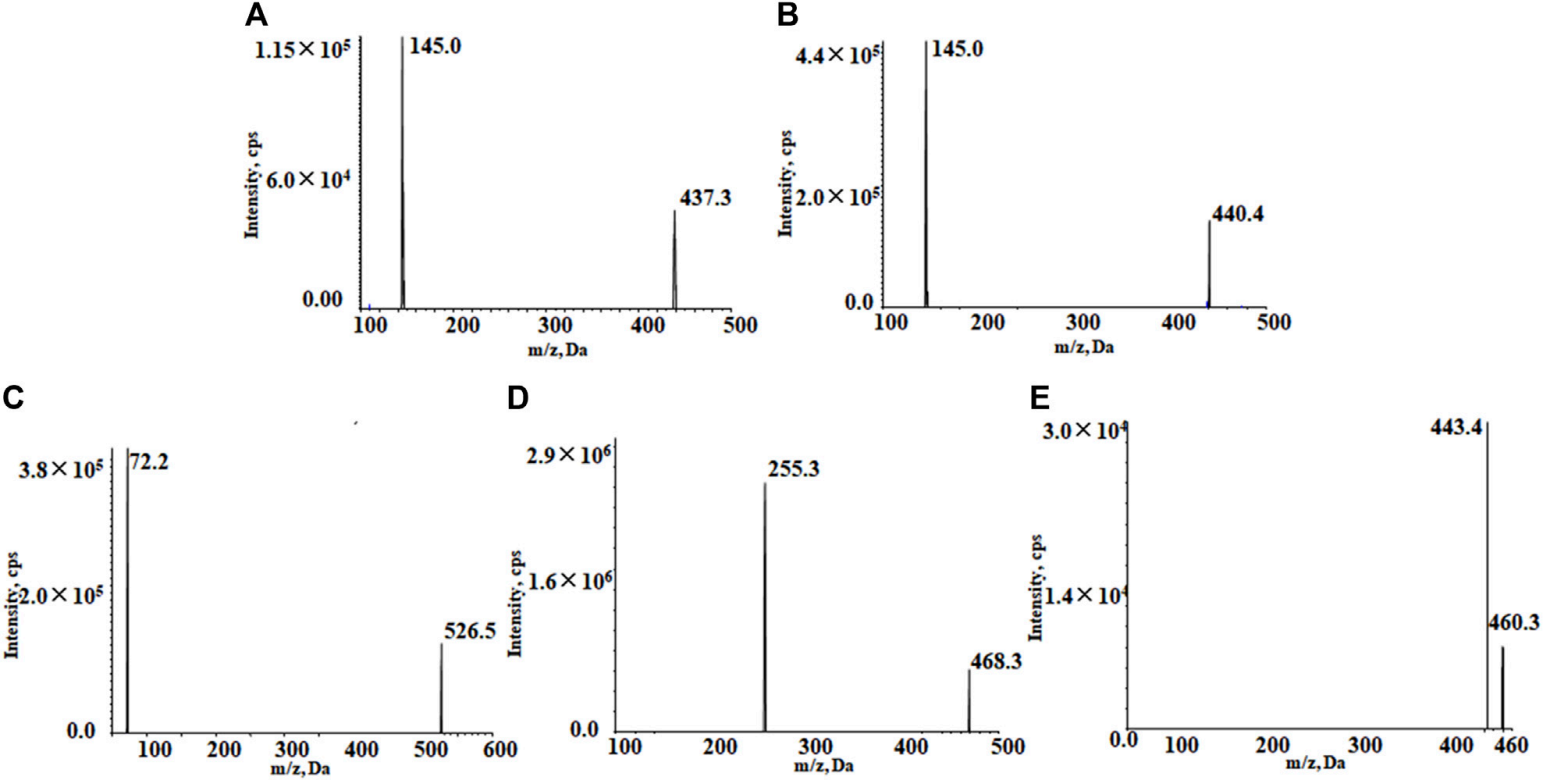
Product ion mass spectrum of rivaroxaban **(A)**, rivaroxaban-d_4_ **(B)**, almonertinib **(C)**, sorafenib-d_3_ **(D)**, and apixaban **(E)**.

The positive ion mode with multiple reaction detection was used. The monitored ion pairs were m/z 526.5→72.2 for almonertinib, 468.3→255.3 for sorafenib-d_3_, 437.3→145.0 for rivaroxaban, 460.3→443.4 for apixaban and 440.4→145.0 for rivaroxaban-d_4_ (Figure 2). The mass spectrometer conditions, including delustering potential (DP) and collision energy (CE) of the compounds, are shown in Table 1. Other parameters of the mass spectrometer were as follows: ion source gas 1, 60.0 psi; ion source gas 2, 50.0 psi; curtain gas, 25.0 psi; source temperature, 500°C; ion spray voltage, 5,500 V.

**TABLE 1:** Experimental setting for the tandem mass-spectrometer for the analytes and internal standards.

Experimental setting	almonertinib	sorafenib-d_3_	rivaroxaban	apixaban	rivaroxaban-d_4_
MRM transition	526.5→72.2	468.3→255.3	437.3→145.0	460.3→443.4	440.4→145.0
Delustering potential (DP), V	120	120	120	80	120
Collision energy (CE), V	80	45	30	38	35
Collision cell exit potential (CXP), V	14	14	14	14	14
Entrance potential (EP), V	10	10	10	10	10

### 2.3 Preparation of stock solution and working solution

Dimethyl sulfoxide was used to make the stock solution with a concentration of 1 mg/mL for almonertinib, rivaroxaban, apixaban, and internal standard (IS). The mixed calibration working solutions were acquired by diluting the stock solution with 50% acetonitrile-water to obtain final concentrations of 5, 20, 50, 100, 500, 1,000, and 2,000 ng/mL (almonertinib); 50, 200, 500, 1,000,5,000, 10,000, and 20,000 ng/mL (rivaroxaban); 10, 50, 100, 500, 1,000, 5,000, and 10,000 ng/mL (apixaban). Quality control (QC) working solutions with concentrations of 10, 800, and 1,500 ng/mL (almonertinib), 100, 8,000, and 15,000 ng/mL (rivaroxaban) and 20, 4,000, and 8,000 ng/mL (apixaban) were prepared using the same method. The mixed IS working solution included 100 ng/mL of sorafenib-d_3_ and 500 ng/mL of rivaroxaban-d_4_, which was also diluted with 50% acetonitrile water. Stock solutions and working solutions were preserved at −20°C and 4°C, respectively.

### 2.4 Preparation of calibration standards and quality control (QC)

The calibration standards were prepared by spiking 5 µL of the mixed working solution with 45 µL of blank rat plasma. The final concentrations of the calibration curves were 0.5, 2, 5, 10, 50, 100, and 200 ng/mL for almonertinib, 5, 20, 50, 100, 500, 1,000, and 2,000 ng/mL for rivaroxaban and 1, 5, 10, 50, 100,500, and 1,000 ng/mL for apixaban. QC samples were obtained by the same method with the final concentrations of 1, 80, and 150 ng/mL for almonertinib, 10, 800 and 1,500 ng/mL for rivaroxaban and 2, 400 and 800 ng/mL for apixaban.

### 2.5 Plasma sample preparation

Protein precipitation was used to process the plasma samples. 5 μL of mixed IS working solution, 50 µL of the plasma samples, and 150 µL of acetonitrile were vortex-mixed for 1.0 min. Then, the mixture was centrifuged at 13,000 × g for 10 min; 100 µL of 50% acetonitrile-water was added to 100 µL of supernatant, vortex-mixed, and centrifuged again. The supernatant was transferred to a 96-well plate for sample analysis.

### 2.6 Method validation

The method was validated according to the guidelines of the Bioanalytical Method Validation Guidance for Industry for the US Food and Drug Administration (US-FDA, 2018) and Chinese Pharmacopoeia (2020). The selectivity, calibration curve linearity, the lower limit of quantification (LLOQ), precision and accuracy, matrix effect, extraction recovery, and stability were assessed during the method validation course.

#### 2.6.1 Selectivity

The selectivity was determined by analyzing blank plasma spiked with a working solution at LLOQ and IS, blank plasma samples from six different batches of rats, and real plasma samples. In the absence of interference, the peak area of blank plasma should be less than 5% of the IS and 20% of the LLOQ within the retention time.

#### 2.6.2 Calibration curve and LLOQ

The calibration curves were evaluated at 0.5–200 ng/mL for almonertinib, 5–2,000 ng/mL for rivaroxaban and 1–1,000 ng/mL for apixaban, respectively. The linearity was determined by the peak area ratios of the analyte to the IS against nominal concentrations using the weighted (1/x^2^) least square linear regression method. The deviation between the back-calculated concentration values calculated from the calibration curve and the theoretical values were acceptable within ± 15%, whereas LLOQ should not surpass 20%.

#### 2.6.3 Precision and accuracy

Precision and accuracy were assessed by analyzing QC samples processed at low, medium, and high concentration levels and LLOQ on three consecutive days. The precision was determined as relative standard deviation (RSD), and the accuracy was expressed by relative error (RE) of six replicates of samples. The precision and accuracy of QC samples were accepted within ± 15%, and LLOQ samples within ± 20%.

#### 2.6.4 Matrix effects and extraction recovery

The matrix effect was evaluated by comparing the analyte peak area in blank plasma samples at low, medium, and high concentrations of QC samples (*n* = 6) with the analyte peak area in the corresponding pure solution. The extraction recovery was assessed by comparing the peak area of the analyte in extracted plasma samples at three concentrations of QC samples (*n* = 6) with the peak area of the analyte of a blank plasma extract spiked at the same concentration.

#### 2.6.5 Stability

The stability of plasma samples was validated by the QC samples at three concentration levels in six replicates under various storage and processing conditions: autosampler for 24 h after preprocessing, room temperature for 8 h, −80°C for 30 days, and three freeze-thaw cycles (−80°C to room temperature). The samples were verified to be stable if the deviation of the samples was not more than ±15% of the standard concentration.

### 2.7 Pharmacokinetic experiments in rats

Healthy male Sprague-Dawley (SD) rats weighing 250 ± 20 g were purchased from Beijing Huafukang Biotechnology Co., Ltd. (Beijing, China). The license number is SCXK (Beijing) 2019–0008. The study was approved and supervised by the Animal Ethics Committee of Hebei General Hospital (Shijiazhuang, China) (No. 2023024). Adaptive feeding (sufficient food and water) was performed for 1 week before the experiment. All the rats were kept under suitable conditions (12 h dark-light diurnal cycle, appropriate temperature at 23°C–25°C, relative humidity of 50% ± 10%). All rats were fasted for 12 h before starting the experiments while still given water.

Sixty healthy rats were randomly divided into ten groups (*n* = 6). Almonertinib was suspended in 0.5% methylcellulose (MC) with 1% DMSO, rivaroxaban was dissolved in 0.5% hydroxypropyl methylcellulose (HPMC), and apixaban was prepared in ultrapure water with 5% DMSO. Group 1 and 2 were treated with the control solvents of almonertinib for nine consecutive days, then with rivaroxaban 2 mg/kg (Group 1) or apixaban 0.5 mg/kg (Group 2) by gavage on the ninth day. Group 3 and 4 were treated with the control solvents of rivaroxaban (Group 3) or apixaban (Group 4) for five consecutive days, then with almonertinib 15 mg/kg by gavage on the fifth day. Group 5–8 received almonertinib 15 mg/kg for nine consecutive days, followed by rivaroxaban, 2 mg/kg (Group 5) or 1 mg/kg (Group 6); apixaban, 0.5 mg/kg (Group 7) or 0.25 mg/kg (Group 8) by gavage on the ninth day. Group 9 and 10 were treated with rivaroxaban 2 mg/kg (Group 9) or apixaban 0.5 mg/kg (Group 10) for five consecutive days, then with almonertinib 15 mg/kg via gavage on the fifth day. Approximately 0.1 mL of blood was collected in a heparinized centrifuge tube via the orbital venous plexus at the following time points: 0, 0.17, 0.34, 0.5, 0.75, 1, 1.5, 2, 3, 4, 5, 6, 8, 10, 12, and 24 h for rivaroxaban; 0, 0.25, 0.5, 1, 1.5, 2, 3, 4, 5, 6, 8, 10, 12, and 24 h for almonertinib; and 0, 0.08, 0.17, 0.25, 0.34, 0.5, 0.75, 1, 3, 5, 7, 10, 12, and 24 h for apixaban. Blood samples were centrifuged at 3,500 *g* for 10 min, and then the supernatant was gathered and stored in a −80°C refrigerator. After, rat intestine, kidney and liver tissues were harvested for molecular analysis on the ninth or fifth day after treatment with the corresponding drugs for each group. The tissues were placed immediately at −80°C.

### 2.8 Quantitative real-time PCR (qRT-PCR) analysis

qRT-PCR analysis was used to determine mRNA levels of Abcb1a, Abcg2 and Cyp3a1 in the liver and intestines, and Abcb1a and Abcg2 in the kidney of rats. Total RNA was isolated from frozen liver, intestine and kidney samples using TRNzol Universal according to the manufacturer’s instructions. A Bio Tek Epoch (Bio Tek Instruments, Inc., Winooski, VT, United States) was employed to quantify the purity and concentration of the total RNA based on the ratio of the absorbance between 260 and 280 nm. RNA samples of 1 µg were transformed to complementary DNA (cDNA) using the FastKing RT Kit. The real-time PCR assays were accomplished using a two-step amplification method based on the recommendations in a SLAN-96S Real-Time PCR system (Shanghai Hongshi Medical Technology Co., Ltd. Shanghai, China). NADPH was used as an internal control, and the PCR cycling criteria were as follows: 95°C for 15 min, then 40 cycles of 95°C for 10, and 60°C for 32 s. The sequences of primers are shown in Table 2.

**TABLE 2:** Primers sequences for qRT-PCR analysis.

Gene	Forward primer	Reverse primer
Abcb1a	5′-TCT​GGT​ATG​GGA​CTT​CCT​TGG​T-3′	5′-TCC​TTG​TAT​GTT​GTC​GGG​TTT​G-3′
Cyp3a1	5′-TGC​ATT​GGC​ATG​AGG​TTT​GC-3′	5′-TTC​AGC​AGA​ACT​CCT​TGA​GGG-3′
Abcg2	5′-TGA​AGA​GTG​GCT​TTC​TAG​TCC​G-3′	5′-TTG​AAA​TTG​GCA​GGT​TGA​GGT​G-3′
NADPH	5′-GCC​TTC​CGT​GTT​CCT​ACC-3′	5′-GCC​TGC​TTC​ACC​ACC​TTC-3′

### 2.9 Statistical analysis

DAS 2.1.1 Software (Mathematical Pharmacology Professional Committee of China, Shanghai, China) was applied to calculate pharmacokinetic parameters using non-compartmental analysis. SPSS 25.0 statistical software (SPSS Inc., Chicago, IL, United States) was applied to statistically analyze the main pharmacokinetic parameters, including AUC, C_max_, T_max_, t_1/2_, CL_z/F_, V_z/F_, and MRT. Statistical comparisons were conducted using a analysis of variance, *t*-test or nonparametric rank-sum test depending on the data type; *p*-value <0.05 was deemed statistically significant.

## 3 Results

### 3.1 Method development and optimization

A UPLC-MS/MS method with high sensitivity and wonderful reproducibility was developed and used to evaluate the pharmacokinetic interactions of almonertinib with rivaroxaban or apixaban in rats. Acetonitrile with 0.1% formic acid was selected as the mobile phase because it showed greater elution capacity than methanol. The addition of 0.1% formic acid to the organic phase B obtained superior peak profiles and minimal background noise. Finally, water (A) and acetonitrile (containing 0.1% formic acid) (B) were chosen as the mobile phase. In general, isotope-labeled internal standards could eliminate mistakes caused by matrix interference and differential ionization properties of the analytes. The internal standard applied to detect almonertinib was a deuterated IS in previous studies (Liu et al., 2022a), but deuterated almonertinib was not readily available. In this study, sorafenib-d_3_ was selected as the IS, which had excellent stability and eliminated the influence of the endogenous matrix. The protein precipitation method has the merits of simplicity, reduced environmental pollution and low cost, compared to liquid-liquid extraction. Acetonitrile was chosen as the protein precipitant.

### 3.2 Method validation

#### 3.2.1 Selectivity

**FIGURE 3:**
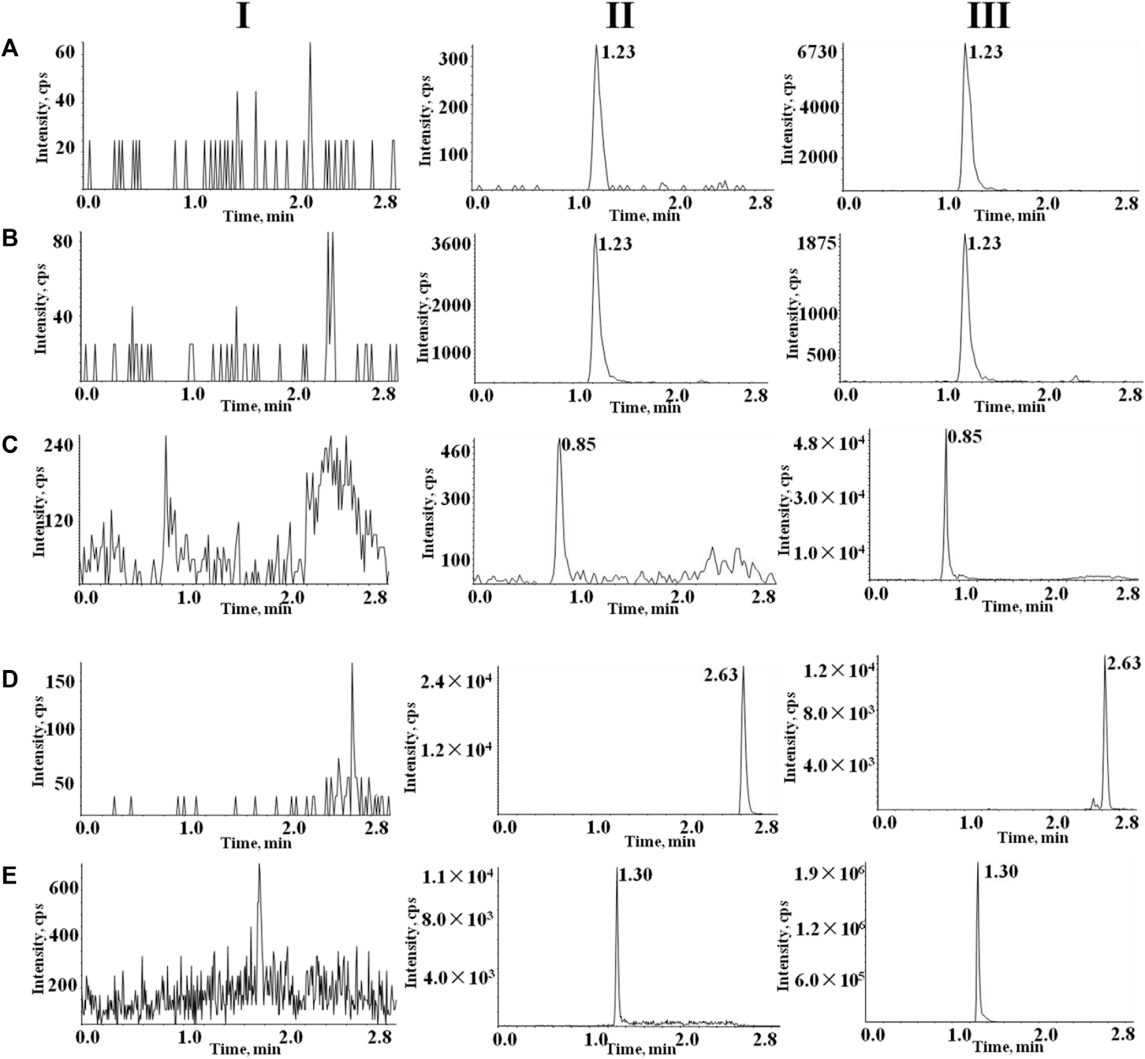
Typical chromatograms of **(A)** rivaroxaban, **(B)** rivaroxaban-d_4_, **(C)** almonertinib, **(D)** and sorafenib-d_3_ **(E)** apixaban. I, blank plasma; II, blank rat plasma with mixed working solution at LLOQ level and IS; and III, rat samples obtained after oral administration of rivaroxaban, apixaban or almonertinib.

Figure 3 shows typical chromatograms of rivaroxaban, rivaroxaban-d_4_, almonertinib, sorafenib-d_3_, and apixaban in different plasma samples. These include blank plasma(I), blank plasma containing the target analytes at LLOQ and internal standard IS (II), and real plasma samples from rats after oral rivaroxaban, apixaban or almonertinib (III). No significant endogenous substance interferences were detected. The retention times for rivaroxaban, rivaroxaban-d_4_, almonertinib, sorafenib-d_3_, apixaban were 1.23, 1.23, 0.85, 2.63 and 1.30 min, respectively.

#### 3.2.2 Calibration curve and LLOQ

Calibration curves were generated using linear regression analysis to concentration ranges of 0.5–200 ng/mL for almonertinib, 5–2,000 ng/mL for rivaroxaban, and,1–1,000 ng/mL for apixaban respectively. The typical calibration curves were Y = 0.373 X + 0.034(r > 0.999) for almonertinib, Y = 0.0651 X + 0.0466 (r = 0.999) for apixaban, and Y = 0.0414 X - 0.0667 (r > 0.999) for rivaroxaban. The LLOQ values for almonertinib, rivaroxaban and apixaban were 0.5, 5, and 1 ng/mL, respectively.

#### 3.2.3 Precision and accuracy

To evaluate accuracy and precision, six replicates of QC samples and LLOQ were analyzed at four concentrations, as shown in Table 3. The intra- and inter-day precision values were no more than 9.4%, and the accuracies ranged from −4.80% to 8.01% for all investigated analyte concentrations in rat plasma. Overall, the precision and accuracy results met the validation requirements.

**TABLE 3:** Intra-day and inter-day precision and accuracy of almonertinib, apixaban and rivaroxaban in rat plasma.

Analytes	Concentration (ng/mL)	Intra-day (n = 6)			Inter-day (n = 18)		
		Mean ± SD	RSD (%)	RE (%)	Mean ± SD	RSD (%)	RE (%)
almonertinib	0.5	0.53 ± 0.04	6.7	6.47	0.51 ± 0.05	9.4	1.83
	1	0.98 ± 0.05	5.4	−2.43	0.98 ± 0.08	8.1	−2.05
	80	77.70 ± 3.27	4.2	−2.88	79.90 ± 4.51	5.6	−0.13
	150	152.50 ± 4.51	3.0	1.67	148.06 ± 7.37	5.0	−1.30
apixaban	1	1.03 ± 0.05	4.6	2.95	0.99 ± 0.06	5.6	−0.21
	2	2.03 ± 0.05	2.7	1.67	2.04 ± 0.07	3.3	1.83
	400	407.17 ± 7.41	1.8	1.79	410.72 ± 15.56	3.8	−3.50
	800	808.50 ± 19.38	2.4	2.13	806.83 ± 22.60	2.8	−1.50
rivaroxaban	5	4.76 ± 0.11	2.3	−4.80	4.93 ± 0.22	4.4	1.29
	10	10.47 ± 0.37	3.6	4.68	10.80 ± 0.42	3.9	8.01
	800	769.00 ± 22.86	3.0	−3.88	779.22 ± 32.08	4.1	−2.60
	1,500	1,486.67 ± 52.41	3.5	−0.89	1,446.11 ± 85.42	5.9	−3.59

#### 3.2.4 Matrix effects and extraction recovery

The matrix effects for almonertinib, apixaban and rivaroxaban ranged from 91% to 96%, from 100% to 108, and from 97% to 103%, respectively (Table 4). The results suggest that endogenous materials had a negligible impact on the analysis. Extraction recovery for almonertinib, apixaban and rivaroxaban in plasma samples was also found to be favorable, with values greater than 96%, 88%, and 100%, respectively. These results demonstrate that protein precipitation using acetonitrile was effective in extracting almonertinib, apixaban, and rivaroxaban. All analytes achieved desirable recoveries, indicating the robustness of this method.

**TABLE 4:** Matrix effects and extraction recovery of almonertinib, apixaban and rivaroxaban in rat plasma (*n* = 6).

Analytes	Concentration (ng/mL)	Matrix effect		Extraction recovery	
		Mean ± SD (%)	RSD (%)	Mean ± SD (%)	RSD (%)
almonertinib	1	92.34 ± 4.94	5.4	104.56 ± 3.01	2.9
	80	91.33 ± 4.94	5.4	100.10 ± 2.00	2.0
	150	96.18 ± 1.82	1.9	96.12 ± 1.54	1.6
apixaban	2	100.38 ± 5.93	5.9	93.31 ± 3.08	3.3
	400	102.36 ± 4.56	4.5	91.01 ± 4.76	5.2
	800	108.06 ± 3.34	3.1	88.40 ± 1.67	1.9
rivaroxaban	10	100.51 ± 1.51	1.5	99.71 ± 2.65	2.7
	800	102.65 ± 2.03	2.0	109.55 ± 1.58	1.4
	1,500	96.74 ± 5.78	6.0	106.73 ± 1.47	1.4

#### 3.2.5 Stability

Stability data for almonertinib, apixaban and rivaroxaban in rat plasma under various storage and processing conditions are summarized in Table 5. The results demonstrate that the plasma samples remained stable for up to 12 h at the auto sampler after processing, for up to 8 h at room temperature, and for up to 30 days at −80°C. Additionally, the samples were stable after undergoing three freeze-thaw cycles (−80°C to room temperature). Both the RE and RSD values were within acceptable limits.

**TABLE 5:** Stability of almonertinib, apixaban and rivaroxaban in rat plasma under different conditions (*n* = 6).

Analytes	Conditions	Concentration (ng/mL)	Mean ± SD (ng/mL)	Precision (RSD%)	Accuracy (RE%)
almonertinib	Autosampler for 12 h	1	0.96 ± 0.05	5.4	−3.88
		80	79.75 ± 4.51	5.7	−0.31
		150	141.00 ± 7.48	5.3	−6.00
	Room temperature for 8 h	1	1.01 ± 0.08	7.7	0.97
		80	80.48 ± 6.42	8.0	0.60
		150	147.50 ± 9.83	6.7	−1.67
	−80°C for 30 days	1	1.02 ± 0.10	10.1	2.33
		80	80.52 ± 3.47	4.3	0.65
		150	146.67 ± 10.63	7.3	−2.22
	Freeze-thaw stability for three times	1	1.01 ± 0.09	8.7	1.47
		80	81.7 ± 2.27	2.8	2.13
		150	146.17 ± 6.68	4.6	−2.56
apixaban	Autosampler for 12 h	2	2.01 ± 0.11	5.5	0.25
		400	423.83 ± 13.30	3.1	5.96
		800	812.17 ± 42.40	5.2	1.52
	Room temperature for 8 h	2	2.08 ± 0.07	3.3	3.83
		400	424.33 ± 9.35	2.2	6.08
		800	804.83 ± 20.23	2.5	0.60
	−80°C for 30 days	2	2.02 ± 0.08	3.8	0.83
		400	400.50 ± 19.99	5.0	0.13
		800	782.83 ± 22.19	2.8	−2.15
	Freeze-thaw stability for three times	2	1.93 ± 0.13	6.8	−3.42
		400	410.67 ± 12.03	2.9	2.67
		800	800.33 ± 24.72	3.1	0.04
rivaroxaban	Autosampler for 12 h	10	10.75 ± 0.48	4.5	7.50
		800	791.33 ± 38.84	4.9	−1.08
		1,500	1,416.67 ± 46.33	3.3	−5.56
	Room temperature for 8 h	10	10.78 ± 0.59	5.4	7.75
		800	833.50 ± 49.92	6.0	4.19
		1,500	1,483.33 ± 72.30	4.9	−1.11
	−80°C for 30 days	10	10.68 ± 0.22	2.1	6.83
		800	765.00 ± 58.14	7.6	−4.38
		1,500	1,435.00 ± 96.07	6.7	−4.33
	Freeze-thaw stability for three times	10	10.11 ± 0.61	6.0	1.13
		800	774.33 ± 23.28	3.0	−3.21
		1,500	1,416.67 ± 48.85	3.5	−5.56

### 3.3 Pharmacokinetic study

#### 3.3.1 Effect of almonertinib on rivaroxaban pharmacokinetics

**FIGURE 4:**
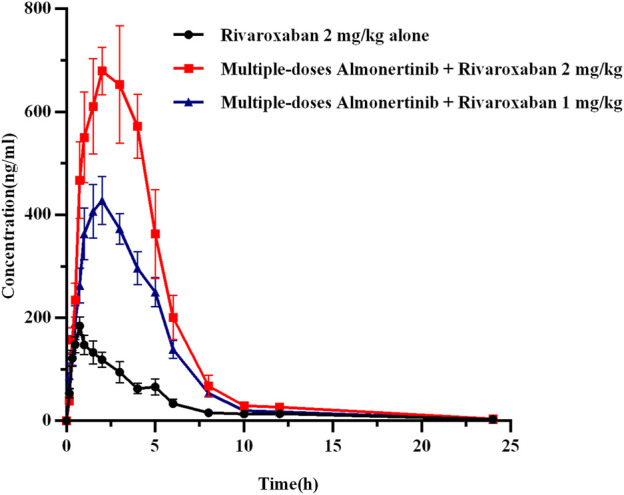
The mean plasma concentration-time profiles of rivaroxaban after oral rivaroxaban alone and following multiple-doses almonertinib. Values are presented as mean ± SD (*n* = 6).

The mean plasma concentration-time curves of rivaroxaban after administration alone and in combination with multiple doses of almonertinib are shown in Figure 4. The main pharmacokinetic parameters of rivaroxaban are summarized in Table 6. The results indicate that multiple doses of almonertinib significantly increased the C_max_, AUC_0-t_, and AUC_0-∞_ of rivaroxaban in group 5 by 3.30, 3.60, and 3.44-fold; rivaroxaban in group 6 by 2.19, 2.06, and 2.02-fold, respectively, compared to the group 1. Additionally, the CL_z_/F of rivaroxaban in group 5 and 6 were significantly decreased by 3.37-fold and 4.57-fold, respectively. The V_z_/F and MRT were also decreased by different extent, and the T_max_ of rivaroxaban in group 5 and 6 were also 1.40-fold (2.21 h) and 1.26-fold (2.08 h) longer than that of the control group (0.92 h). However, there were no statistically significant changes in the t_1/2z_ between the groups.

**TABLE 6:** Pharmacokinetic parameters of rivaroxaban in rats after oral administration alone and combined multiple doses of almonertinib.

Parameters (Unit)	Rivaroxaban (2 mg/kg, group 1)	Multiple-doses almonertinib with rivaroxaban (2 mg/kg, group 5)	Multiple-doses almonertinib with rivaroxaban (1 mg/kg, group 6)
AUC_0-t_ (μg/L*h)	763.89 ± 164.17	3,511.15 ± 783.84**	2,216.88 ± 455.02**
AUC_0-∞_(μg/L*h)	796.70 ± 168.72	3,536.15 ± 793.55**	2,234.87 ± 464.38**
C_max_ (μg/L)	200.50 ± 37.99	862.00 ± 110.34**	457.33 ± 101.03**
T_max_ (h)	0.92 ± 0.30	2.21 ± 0.95**	2.08 ± 0.49**
t_1/2z_ (h)	6.84 ± 3.46	4.47 ± 0.80	4.30 ± 0.91
CL_z_/F (L/h/kg)	2.62 ± 0.62	0.60 ± 0.18**	0.47 ± 0.12**
V_z_/F (L/kg)	26.35 ± 18.02	3.75 ± 0.72**	2.83 ± 0.55**
MRT_0-t_ (h)	4.72 ± 0.57	3.94 ± 0.56*	4.10 ± 0.41
MRT_0-∞_ (h)	6.09 ± 1.45	4.12 ± 0.60**	4.30 ± 0.47**

*p < 0.05, **p < 0.01, compared with 2 mg/kg rivaroxaban alone, demonstrating statistically significant difference. The main pharmacokinetic parameters are revealed as the mean ± standard deviation.

#### 3.3.2 Effect of almonertinib on apixaban pharmacokinetics

**FIGURE 5:**
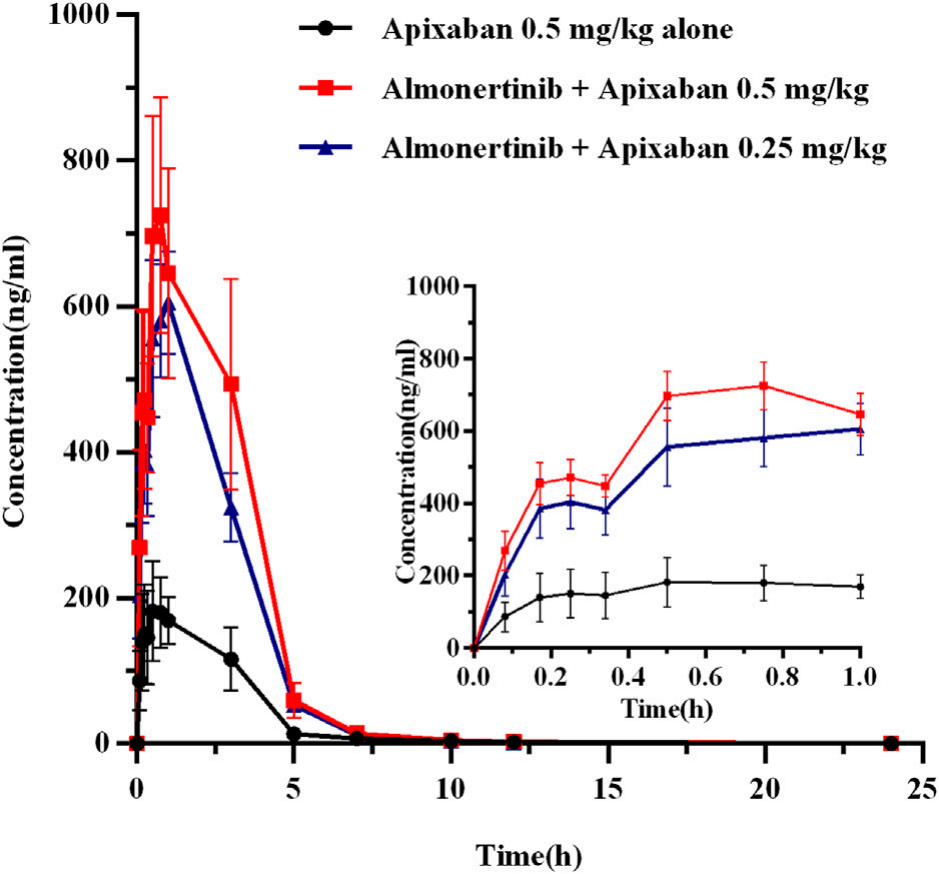
The mean plasma concentration-time profiles of apixaban after oral apixaban alone and following multiple-doses almonertinib. Values are presented as mean ± SD (*n* = 6).

The mean plasma concentration-time curves of apixaban after administration alone and following multiple doses of almonertinib are shown in Figure 5. The main pharmacokinetic parameters of apixaban are summarized in Table 7. Compared with the apixaban 0.5 mg/kg alone group (group 2), the C_max_, AUC_0-t_, and AUC_0-∞_ of apixaban in group 7 were significantly increased by 2.69, 2.87, and 2.82-fold; apixaban in group 8 were significantly increased by 2.19, 2.06, and 2.02-fold, respectively. Additionally, the CL_z_/F of apixaban in group 7 and 8 were significantly decreased by 3.00-fold and 5.29-fold, respectively. The V_z_/F, MRT_0-∞_ and t_1/2z_ were also decreased by different extent. However, there were no statistically significant changes in the T_max_ and MRT_0-t_ between the groups.

**TABLE 7:** Pharmacokinetic parameters of apixaban in rats after oral administration alone and combined multiple doses of almonertinib.

Parameters (Unit)	Apixaban (0.5 mg/kg, group 2)	Multiple-doses almonertinib with apixaban (0.5 mg/kg, group 7)	Multiple-doses almonertinib with apixaban (0.25 mg/kg, group 8)
AUC_0-t_ (μg/L*h)	611.25 ± 167.75	2,363.74 ± 495.25**	1867.44 ± 528.72**
AUC_0-∞_(μg/L*h)	620.53 ± 167.65	2,369.49 ± 496.36**	1874.21 ± 527.67**
C_max_ (μg/L)	202.33 ± 50.85	746.83 ± 158.83**	645.00 ± 190.97**
T_max_ (h)	0.68 ± 0.28	0.63 ± 0.14	0.79 ± 0.25
t_1/2z_ (h)	2.95 ± 0.96	1.81 ± 0.23**	2.17 ± 0.15*
CL_z_/F (L/h/kg)	0.88 ± 0.35	0.22 ± 0.05**	0.14 ± 0.04**
V_z_/F (L/kg)	3.72 ± 1.63	0.58 ± 0.20**	0.44 ± 0.11**
MRT_0-t_ (h)	2.19 ± 0.13	2.08 ± 0.13	2.01 ± 0.32
MRT_0-∞_ (h)	2.42 ± 0.16	2.11 ± 0.13*	2.06 ± 0.36*

*p < 0.05, **p < 0.01, compared with 0.5 mg/kg apixaban alone, demonstrating statistically significant difference. The main pharmacokinetic parameters are revealed as the mean ± standard deviation.

#### 3.3.3 Effect of rivaroxaban or apixaban on almonertinib pharmacokinetics

**FIGURE 6:**
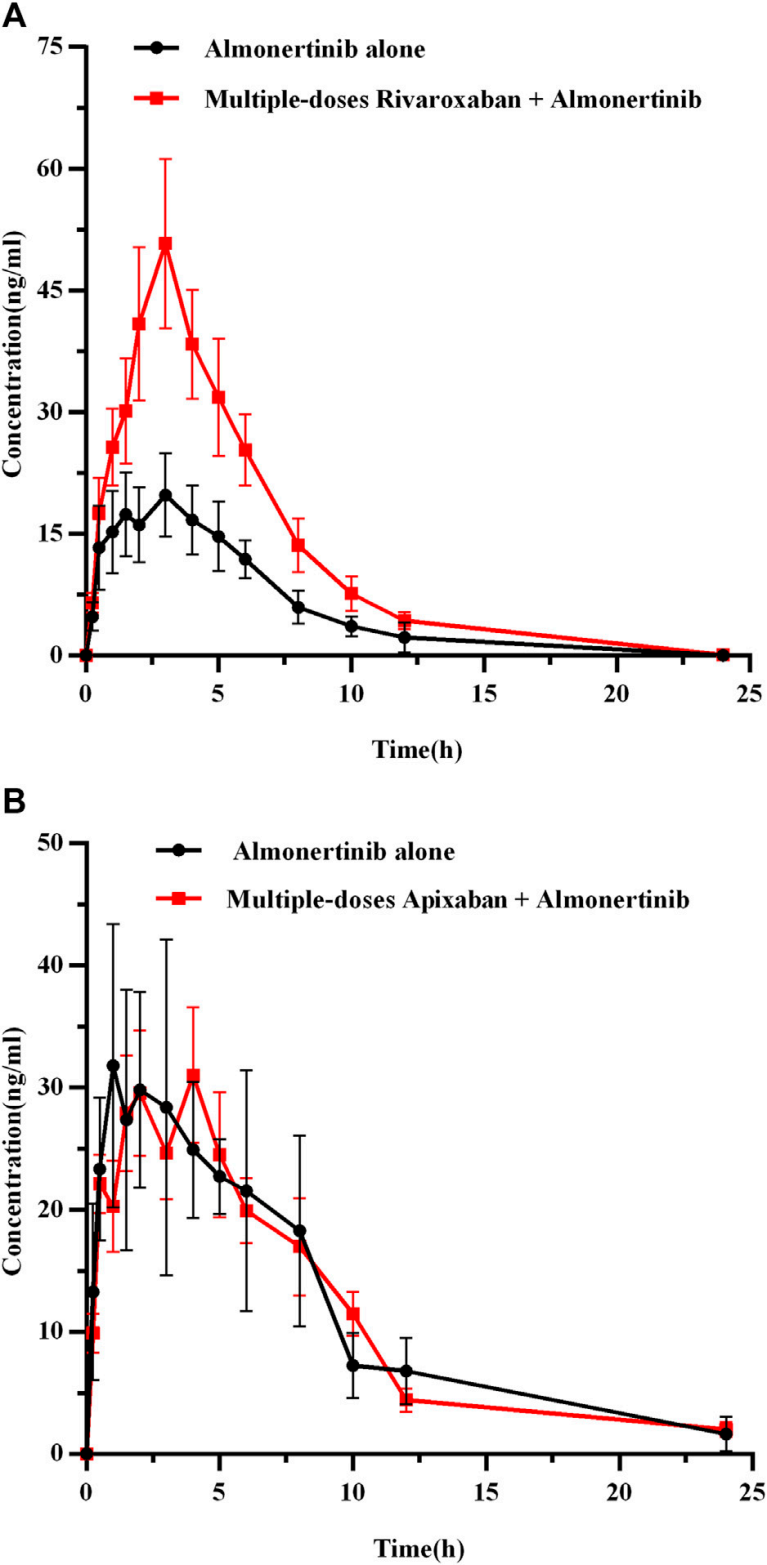
The mean plasma concentration-time profiles of almonertinib after oral almonertinib alone and following multiple-doses rivaroxaban **(A)** or apixaban **(B)**. Values are presented as mean ± SD (*n* = 6).

The mean plasma concentration-time curves for almonertinib (15 mg/kg) when administered alone and after multiple doses of rivaroxaban (2 mg/kg) or apixaban (0.5 mg/kg) are presented in Figure 6, and the major pharmacokinetic parameters of almonertinib are summarized in Table 8. Compared with the almonertinib 15 mg/kg alone (group 3), the C_max_, AUC_0-t_, and AUC_0-∞_ of almonertinib was significantly increased by 1.26, 1.17 and 1.17-fold, respectively, when administered after multiple doses of rivaroxaban. Furthermore, the CL_z_/F of almonertinib decreased significantly by 81.1%. However, other pharmacokinetic parameters, including T_max_, t_1/2z_, V_z_/F, MRT_0-t_, and MRT_0-∞_, showed no significant difference between group 3 and 9. In addition, all the pharmacokinetic parameters of almonertinib in group 10 showed no statistically significant difference compared with group 4.

**TABLE 8:** Pharmacokinetic parameters of almonertinib in rats after oral administration alone and combined multiple doses of rivaroxaban or apixaban.

Parameters (Unit)	Almonertinib (15 mg/kg)			
	Alone (group 3)	With multiple-doses rivaroxaban (group 9)	Alone (group 4)	With multiple-doses Apixaban(group 10)
AUC_0-t_ (μg/L*h)	138.29 ± 31.29	299.48 ± 149.69*	280.58 ± 43.26	266.75 ± 51.89
AUC_0-∞_(μg/L*h)	138.29 ± 31.29	299.48 ± 149.69*	298.74 ± 46.78	292.64 ± 48.88
C_max_ (μg/L)	22.77 ± 4.76	51.43 ± 24.91**	39.82 ± 8.12	42.15 ± 5.99
T_max_ (h)	2.17 ± 1.13	3.17 ± 0.41	1.83 ± 1.03	2.50 ± 1.18
t_1/2z_ (h)	2.18 ± 1.05	2.70 ± 0.78	5.40 ± 4.96	6.79 ± 4.15
CL_z_/F (L/h/kg)	112.62 ± 22.42	62.17 ± 31.01**	51.29 ± 8.37	52.59 ± 9.62
V_z_/F (L/kg)	330.81 ± 125.45	243.80 ± 167.75	383.29 ± 315.50	522.18 ± 317.78
MRT_0-t_ (h)	5.03 ± 0.73	5.15 ± 0.66	6.53 ± 0.97	6.49 ± 0.35
MRT_0-∞_ (h)	5.03 ± 0.74	5.15 ± 0.66	8.35 ± 3.38	9.31 ± 2.59

*p < 0.05, **p < 0.01, compared with 15 mg/kg almonertinib alone, demonstrating statistically significant difference. The main pharmacokinetic parameters are revealed as the mean ± standard deviation.

### 3.4 mRNA expression in the liver, intestines and kidney

**FIGURE 7:**
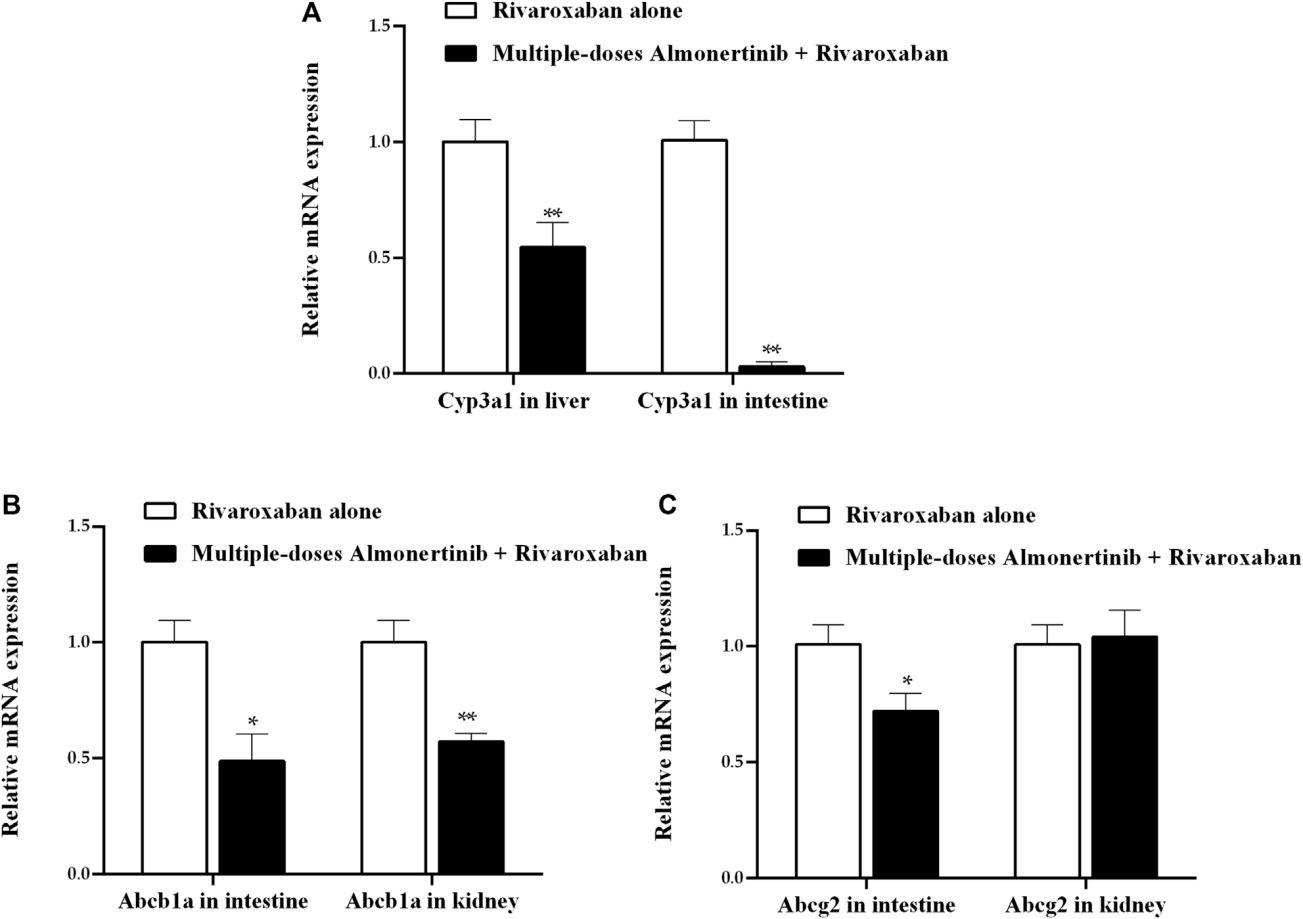
Relative expression ratios of mRNA for Cyp3a1 in liver and intestine, Abcb1a, Abcg2 in intestine and kidney of rats. **(A)** Effect of multiple-dose almonertinib treatment on mRNA expression of Cyp3a1 in liver and intestine. **(B)** Effect of multiple-dose almonertinib treatment on mRNA expression of Abcb1a in intestine and kidney. **(C)** Effect of multiple-dose rivaroxaban treatment on mRNA expression of Abcg2 in intestine and kidney. Values are presented as mean ± SD (*n* = 3). *p < 0.05, **p < 0.01.

To investigate the possible mechanism underlying the pharmacokinetic interactions between almonertinib and rivaroxaban or apixaban involving transporters and metabolic enzymes, we assessed the mRNA expression of CYP3A4 (Cyp3a1) in liver and intestine, and P-gp (Abcb1a), BCRP (Abcg2) in intestine and kidney of rats. The results, as shown in Figure 7, indicate that almonertinib administered to rats for nine consecutive days significantly inhibited the mRNA expression of Cyp3a1 by 45.5% in the liver and 96.9% in the intestine. Almonertinib also inhibited the mRNA expression of Abcb1a and Abcg2 by 51.3% and 28.0% in the intestine. Moreover, the mRNA expression of Abcb1a was inhibited by 42.9% in kidney.

## 4 Discussion

Anomalous hemodynamics and physiological disorders of cancer patients result in a high incidence of thrombotic diseases. Anticoagulant therapy is frequently indicated for lung cancer patients, who often require long-term and multiple pharmacological treatments. However, combining multiple drugs can generate drug interactions in both pharmacodynamics and pharmacokinetics. A comprehensive evaluation has shown that the number of potential DDIs significantly increases with the number of comorbidities and drugs used in cancer patients (Koni et al., 2022). This can impact treatment outcomes by increasing the incidence of drug-related adverse reactions or reducing efficacy. For example, a previous study (Scholz et al., 2021) showed that *H. perforatum* extracts reduced rivaroxaban exposure and the pharmacodynamic effect. Another retrospective cohort study (Hanigan et al., 2020) found that patients receiving rivaroxaban with amiodarone, dronedarone, diltiazem, or verapamil had an increased risk of bleeding in the real world. Wen et al. (Wen et al., 2022) showed that co-administration with dronedarone increased the exposure of apixaban and might enhance major bleeding risks. Therefore, it is crucial to identify potential DDIs to minimize risks of unanticipated outcomes.

This study developed and validated a simple and reliable method for measuring almonertinib, rivaroxaban and apixaban in rat plasma. The developed method was successfully used to investigate pharmacokinetic interactions between almonertinib and rivaroxaban or apixaban in rats. To mimic the recommended doses for patients in clinical practice, oral dosages of 15 mg/kg for almonertinib, 2 mg/kg for rivaroxaban and 0.5 mg/kg for apixaban were used in rats (Reagan-Shaw et al., 2008). The half-life for almonertinib was approximately 30–35 h (Yang et al., 2020), for apixaban was approximately 12 h (Byon et al., 2019), while the terminal half-life for rivaroxaban ranged from 5–9 h in healthy young subjects to 11–13 h in elderly subjects (Kubitza et al., 2005a; Kubitza et al., 2008). Because it takes 5-7 half-lives for a drug to reach steady-state blood levels *in vivo*, almonertinib was orally administered for 9 days, and rivaroxaban and apixaban were orally administered for 5 days, respectively, to reach a steady-state concentration of the three drugs in the rats.

Co-administration of rivaroxaban with almonertinib for multiple doses resulted in an increase in the C_max_ and AUC of rivaroxaban by more than 3.0-fold, along with an obvious decrease in CL_z_/F and V_z_/F were observed. Multiple doses of almonertinib significantly increased the C_max_ and AUC of apixaban by close to 3.0-fold, and reduced the CL_z_/F. Thus, it can be hypothesized that almonertinib increases rivaroxaban and apixaban absorption and/or inhibits their metabolism. In this study, we speculated that the mechanism leading the systemic exposure of rivaroxaban and apixaban increase was absorption enhancement. Rivaroxaban and apixaban are substrates of the efflux transporter P-gp and BCRP, which modulate absorption in the small intestines, as well as CYP3A4 which facilitate metabolism in the small intestines and liver. The literature indicated that inhibition of CYP3A4 and P-gp can lead to increase in the systemic exposure of rivaroxaban and apixaban. Kim et al. (Kim et al., 2019) used a pharmacokinetic/pharmacodynamics modeling to explore the influence of verapamil (a inhibitor of P-gp and CYP3A4) on rivaroxaban. The result showed that verapamil increased the systemic exposure of rivaroxaban by 2.8-fold. A research (Frost et al., 2015) showed that a 2.0-fold and 1.4-fold increase in exposure to apixaban was observed when combined with ketoconazole and diltiazem, respectively. Apart from being substrates for P-gp and CYP3A4, rivaroxaban and apixaban are also substrates for BCRP. BCRP showed a higher affinity than P-gp, the role of BCRP among the DDIs related to rivaroxaban and apixaban cannot be excluded (Zhao et al., 2022). We found that almonertinib significantly inhibited the mRNA levels of CYP3A4, P-gp and BCRP in the intestines, which may lead to decreased drug efflux and metabolism in the small intestines, resulting in increased bioavailability. In addition to oral absorption, the metabolism of the drugs also related to systemic exposure. We speculated that metabolism inhibition may not be the major reason for increased exposure of rivaroxaban and apixaban in rats, for the reason of the t_1/2_ of rivaroxaban has been observed with no significant changes, whereas the t_1/2_ of apixaban is decreased, although mRNA expression of CYP3A4 in the liver was inhibited. In the current study, our finding confirmed almonertinib significantly enhanced the systemic exposure of rivaroxaban and apixaban in rats, and the mechanism might be almonertinib induced by the absorption of the two drugs in the intestines. However, the increased exposure to rivaroxaban and apixaban may be connected with an increased risk of major bleeding. Additionally, when almonertinib is combined with other drugs that are substrates for CYP3A4, P-gp or BCRP in NSCLC patients, special attention to DDIs attributed to this inhibitory effect is essential.

It is worth noting that rivaroxaban is both a victim and a perpetrator in this study. We found that co-administrating almonertinib with multiple doses of rivaroxaban could significantly increase C_max_ and AUC, which significantly decreases CL_z_/F. These findings are not in line with previous research, which indicated that there was no clinically relevant pharmacokinetic drug interaction between rivaroxaban and the CYP3A4 substrate midazolam, the P-gp substrate digoxin, or with the CYP3A4/P-gp substrate atorvastatin(Kubitza et al., 2012), as demonstrated in phase I studies and *in vitro* research (Mueck et al., 2013). Additionally, almonertinib and rivaroxaban are both substrates for CYP3A4 and P-gp, and the two drugs may compete for the same metabolic enzyme or transporters, resulting in decreased metabolic clearance and increased blood levels of almonertinib. Overall, the changes in pharmacokinetic parameters of almonertinib observed in this study can be explained by the reasons referred to above, but many assumptions require further research.

Based on the above findings, the effects of lowering the dose on the blood concentration of rivaroxaban and apixaban were investigated in depth. Our study found that after a half dose reduction of rivaroxaban, multiple doses of almonertinib increased the C_max_ and AUC of rivaroxaban by approximately 1.3 and 1.9-fold. Correspondingly, after the oral multiple doses of almonertinib, the C_max_ and AUC of apixaban increased by 2.2 and 2.1-fold, when the dose of apixaban was reduced by half. Hence, when almonertinib is used in combination with rivaroxaban or apixaban in clinical practice, it is recommended to monitor of coagulation indicators such as prothrombin time at the maximal blood concentration, which is approximately 2–3 h (±1 h) after take orally for each of two drugs (Steffel et al., 2021), and use indexs as a basis for appropriate dose adjustment. In addition, physicians and pharmacists should monitor patients closely, especially those with renal insufficiency, in which drug excretion is reduced and drug interactions may lead to even less excretion, to prevent potential adverse drug reactions. Notably, exposure to rivaroxaban and apixaban did not decrease proportionally with dose reduction.

This study has several limitations. First, many of the mechanisms mentioned in the article are mostly inferred and require further study. Second, animal models with lung cancer or thromboembolism were not used to examine pharmacokinetic interactions. Third, studies have shown that CYP2J2 has a higher catalytic efficiency than CYP3A4, but our study did not investigate the effect of almonertinib on CYP2J2. Fourth, species differences exist between rats and humans exist, and the drug interactions we observed in rats may not necessarily occur in humans. Therefore, further studies in clinical settings are needed to confirm whether similar interactions occur in humans.

## 5 Conclusion

In this study, we developed and validated a rapid method for the simultaneous determination of almonertinib, rivaroxaban and apixaban in rats. The method was successfully applied to investigate the pharmacokinetic interaction between the three drugs. Based on the experiments we speculate that almonertinib significantly increased systemic exposure to rivaroxaban and apixaban by inhibiting CYP3A4, BCRP and P-gp. Similarly, co-administration of almonertinib with multiple doses of rivaroxaban increased the bioavailability of almonertinib. The pharmacokinetic results suggest that active surveillance for adverse drug reactions should be conducted when the drugs are combined in clinical practice. However, further verification is necessary through clinical trials.

## Data Availability

The original contributions presented in the study are included in the article/Supplementary Material, further inquiries can be directed to the corresponding author.
